# GSH-Activatable Metal-Phenolic Networks for Photothermal-Enhanced Chemotherapy and Chemodynamic Therapy

**DOI:** 10.3390/jfb14090436

**Published:** 2023-08-23

**Authors:** Weijun Chen, Meiyang Yang, Jie Li, Zhilan Chen, Lefei Hu, Jiannan Zhang, Liangyu Cai, Lipeng Qiu, Jinghua Chen

**Affiliations:** 1 School of Life Sciences and Health Engineering, Jiangnan University, Wuxi 214122, China; weijunchenaiwhl@outlook.com (W.C.); y13002666962@163.com (M.Y.); 6211507007@stu.jiangnan.edu.cn (J.L.); 6211504001@stu.jiangnan.edu.cn (Z.C.); 2 School of Chemical and Material Engineering, Jiangnan University, Wuxi 214122, China; 3 Department of Biological Science, National University of Singapore, Singapore 119077, Singapore; leminhy@sina.com; 4 Department of Anesthesiology, Wuxi Traditional Chinese Medicine Hospital, Wuxi 214071, China; zjn_wxzy@126.com (J.Z.); cailiangyu2110@163.com (L.C.)

**Keywords:** metal-phenolic networks, GSH-activatable, photothermal therapy, chemodynamic therapy, chemotherapy

## Abstract

Chemotherapy (CT) plays an important role in the antitumor process, but the unsatisfactory therapeutic efficacy and the obvious toxic side effects of CT seriously restrict its application. To overcome the limitations of CT, the strategy of chemotherapy enhanced by chemodynamic therapy (CDT) and photothermal therapy (PTT) has been considered a promising approach to improve the anticancer effect. Herein, a novel GSH-activatable Cu^2+^-Quercetin network (QC) was synthesized via a convenient strategy to load Au nanoparticles (NPs) and DOX, named QCDA, for the synergistic therapy of CT/CDT/PTT. The results showed that QCDA exhibited GSH-sensitive degradation and “cargos” release in cancer cells, and then PTT and CDT caused by Au NPs and Cu^+^ significantly enhanced the CT effect of DOX and Quercetin on anticancer. More importantly, the PTT and depleted GSH accelerated the Fenton-like ionization process resulting in facilitating the CDT efficiency. Collectively, the multi-mode synergistic strategy of CT/CDT/PTT, which showed an excellent therapeutic effect, maybe a potential therapeutic pathway for anticancer.

## 1. Introduction

Chemotherapy (CT) has been widely used for cancer treatments [1,2,3]. Doxorubicin (DOX), one of the first-line drugs for cancer therapy, can be embedded into the DNA double helix to inhibit the topoisomerase I activity and promote cancer cells’ death [4]. However, the use of DOX alone can cause obvious side effects (especially heart failure [5]), susceptible drug resistance and damage to the immune system [6,7]. As a result, synergistic therapy has emerged as a new strategy to reduce the side effects of chemical drugs and improve their antitumor effect.

Chemodynamic therapy (CDT), which catalyzes hydrogen peroxide (H_2_O_2_) to produce highly reactive oxygen species (ROS) in cancer cells through the Fenton reaction, has been extensively studied [8,9]. The activity of the Fenton catalyst is one of the key factors of the CDT effect. Transition metals of the fourth period have been extensively studied as Fenton catalysts, such as Mn^2+^ [10], Fe^2+^ [11] and Cu^+^ [12]. Compared with Mn^2+^ and Fe^2+^, Cu^+^ exhibited optimal Fenton catalytic activity in both weakly acidic and neutral conditions [13]. In addition, the Fenton reaction efficiency was also limited by the reaction temperature. Therefore, increasing the temperature of the tumor site during CDT was a promising strategy.

Photothermal therapy (PTT), utilizing photothermal agents to increase the temperature under near-infrared (NIR) light irradiation to result in cancer cell damage, has emerged as a promising approach for cancer treatment [14,15,16,17]. On the other hand, the increase in cancer tissue temperature can also enhance the efficiency of the Fenton catalyst, which contributes to the faster generation of highly toxic ROS for CDT [18,19,20]. Thus far, a variety of nanomaterials have been reported for PTT, such as carbon nanotubes [21], copper sulfide [22], graphene [23] and gold nanoparticles (Au NPs) [24,25], in which Au NPs attracted more attention due to their excellent biocompatibility, desirable near-infrared (NIR) absorption, well-controlled sizes and shapes and ideal photothermal conversion efficiency [26,27,28].

The multi-mode synergistic therapy was a widely recognized and effective treatment for anticancer, but the construction of a stimuli-responsive drug delivery system (DDS) by “all in one” strategy for the synergistic therapy of CT, CDT and PTT still remained challenging. Metal-phenolic networks, built from metal ions or clusters coordinated with polyphenol, were a potential DDS in the field of synergistic therapy [29,30,31]. Quercetin (3,3′,4′,5′-7-pentahydroxy-flavone), one of the most abundant flavonoids in plants [32,33], has been reported to have antitumor effects, which can coordinate with a variety of metals to form metal-phenolic networks [34]. Therefore, metal-Quercetin networks can be used not only as a nanocarrier but also as a traditional Chinese medicine with anticancer activity. In addition, the stimulus-response should also be given to the DDS in order to specifically release “cargo” at the tumor site based on the special tumor microenvironments (TME), such as lower pH, high expression of glutathione (GSH) and high concentration of adenosine triphosphate (ATP) [35,36]. GSH-responsive DDS, which was attacked and broken down by the reduced GSH, played a dual important role in GSH decomposition and targeted release. 

**Scheme 1:**
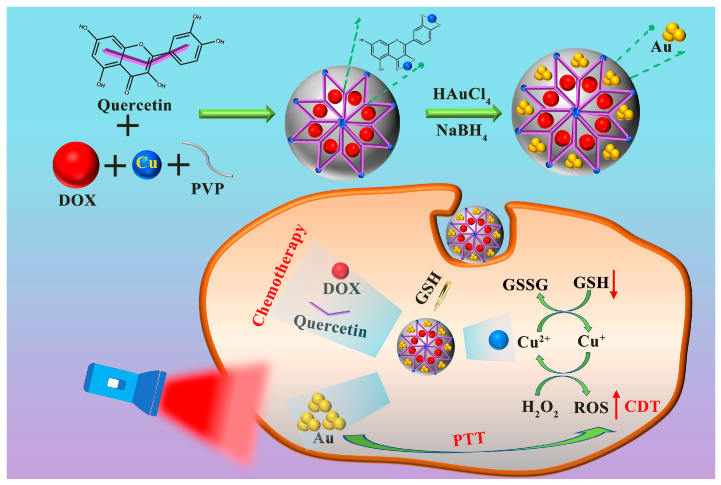
Schematic illustration of the synthesis and synergistic antitumor therapy of QCDA.

Herein, we synthesized GSH-responsive Quercetin-Cu^2+^ (QC) networks loading with DOX and Au NPs (QCDA). The nanotheranostics system was designed based on the following considerations (as shown in Scheme 1): (1) Quercetin can not only serve as the skeleton of the DDS but also maintain its own traditional Chinese medicine activity for anticancer [34]. (2) QC networks can be decomposed by reducing Cu^2+^ to Cu^+^ by GSH to release DOX in cancer cells for mitigating the side effects of chemotherapy [37]. (3) The reduced Cu^+^ can act as a highly efficient Fenton catalyst to decompose H_2_O_2_ and produce ROS for CDT [38]. (4) Au NPs can increase the temperature of the tumor site for PTT and the increased temperature can also improve the catalytic activity of Cu^+^ for the enhanced CDT [39,40]. By integrating DOX-induced CT, Cu^2+^ reduction-enhanced CDT and Au NP-induced PTT and enhanced CDT, the prepared QCDA became a promising nanotheranostic for CT, CDA and PTT synergistic anticancer therapy.

## 2. Materials and Methods

### 2.1. Materials

All reagents were purchased and used without further purification. Quercetin (Que) was purchased from Sun Chemical Technology Co., Ltd. (Shanghai, China). Copper(II) acetate monohydrate was purchased from Shanghai Titan Technology Co., Ltd. The reduced glutathione (GSH), H_2_O_2_ Quantitative Assay Kit (Water-Compatible) and 3-(4,5-Dimethyl-2-Thiazolyl)-2,5-Diphenyl Tetrazolium Bromide (MTT) were purchased from Sangon Biotech Co., Ltd. (Shanghai, China). 2′,7′-dichlorodihydrofluorescein diacetate (DCFH-DA) was purchased from Absin Bioscience Inc. (NaBH_4_), tetrachloroauric (III) acid tetrahydrate (HAuCl_4_·4H_2_O), N,N-Dimethylformamide (DMF, AR), polyvinyl pyrrolidone (PVP) and hydrogen peroxide (H_2_O_2_) were purchased from Sinopharm Chemical Reagent Co., Ltd. (Shanghai, China).

### 2.2. Synthesis of QC

QC was synthesized following the previous literature with slight modifications [34]. In detail, 6 mL of DMF solution containing 0.2 mmol Quercetin (60.4 mg) and PVP (40 mg) was mixed with 6 mL of Copper (II) acetate monohydrate (0.4 mmol, 72.7 mg) DMF solution. After being stirred for 10 min, the coordination compounds of copper and Quercetin obtained by centrifugation (10,000 rpm, 10 min) were washed twice with deionized water and then redispersed in deionized water (3 mg/mL) for further use. All of the above processes were conducted at room temperature.

### 2.3. Preparation of QCD

The synthesis of QCD is very similar to that of QC. Briefly, 6 mL of DMF solution was mixed with 0.2 mmol Quercetin (60.4 mg), 0.05 mmol DOX and 40 mg PVP and was stirred for 30 min. Then, the Copper (II) acetate monohydrate (0.4 mmol, 72.7 mg) DMF solution (6 mL) was dropwise added to the above solution and stirred for 2 h. The resulting DOX-loaded nanoparticles were obtained after centrifugation and washed with deionized water three times, and then redispersed in deionized water (3 mg/mL) for further use.

### 2.4. Au Nanoparticles Loading

The QCD solution (6 mL, 3 mg/mL) was placed in an ice-water bath under stirring, followed by the addition of 300 μL of 30 mM HAuCl_4_ and stirring for 5 min. Thereafter, 450 μL of freshly prepared ice-cold NaBH_4_ solution (30 mM) was added and stirred for 1 min, followed by washing with deionized water twice (named QCDA) and dispersion in deionized water (3 mg/mL) [41] for further use.

### 2.5. Characterizations of DOX, Quercetin, QC, QCD and QCDA

The FT-IR spectra of DOX, Quercetin, QC, QCD and QCDA were obtained using a Fourier transform infrared spectrometer (FTIR, Nicolet 6700, Bruker, Karlsruhe, Germany). The UV−vis spectral data of Quercetin, QC, QCD and QCDA were captured using a spectrophotometer (UV-2550, SHIMADZU, Kyoto, Japan). The surface chemistry of QCDA was investigated by energy-dispersive X-ray spectroscopy (EDS, S-4800, Hitachi, Tokyo Met., Japan). The hydrodynamic size and zeta potential of QC, QCD and QCDA were measured by dynamic light scattering (DLS, Zetasizer Nano ZS, Malvern Ltd., Worcestershire, UK). The morphology of QC, QCD and QCDA were obtained using scanning electron microscopy (SEM, Regulus8100, Hitachi, Tokyo Met., Japan).

### 2.6. pH/GSH Response of QC

A total of 10 mg of QC was added to the solutions of PBS 7.4, PBS 5.0, 10 mM GSH and PBS 5.0 with 10 mM GSH and stirred for 24 h. Then, the QC solution was centrifuged (13,000 rpm, 10 min) and washed three times with water. The precipitate was freeze-dried and used for FT-IR testing.

### 2.7. pH/GSH Response of QCD

A total of 200 μg of QCD was added to 4 mL of PBS 7.4, PBS 5.0, and PBS 5.0 containing 10 mM GSH under stirring for 12 h. A total of 200 μg of QC was added to 4 mL of PBS 7.4 as contrast. The fluorescence intensity of the supernatant obtained by centrifugation (8000 rpm, 5 min) was observed by a spectrofluorophotometer, and the excitation wavelength was 480 nm. (RF-6000, SHIMADZU, Kyoto, Japan).

### 2.8. In Vitro Drug Release Study 

The release behaviors of QCDA were investigated using the dialysis method. The physiological environment and tumor intracellular environment were simulated by PBS 7.4 and PBS 5.0 with or without 10 mM GSH. A total of 400 μL of QCD solutions (3.75 mg/mL) was transferred into a dialysis bag and immersed in 20 mL of different PBS at 37 °C. A total of 2 mL of the solutions was taken out and replenished with 2 mL of corresponding fresh PBS. The release behaviors of QCD were evaluated by a spectrofluorophotometer.

### 2.9. H_2_O_2_ Depletion with QC, QCD and QCDA

A total of 1 mL of H_2_O_2_ solution (2 mM) with or without GSH (20 mM) was mixed with QC solution (1 mL, 3 mg/mL), QCD solution (1 mL, 3 mg/mL) and QCDA solution (1 mL, 3 mg/mL). After stirring for 30 min at 37 °C, the supernatant obtained by centrifugation (13,000 rpm, 10 min) was used to detect the remaining H_2_O_2_ according to the H_2_O_2_ Quantitative Assay Kit.

### 2.10. Extracellular Chemodynamic Activity of QC, QCD and QCDA

3,3′,5,5′-Tetramethylbenzidine (TMB) was used to detect •OH, which can be oxidized to the oxidation state of TMB (signal peak at 652 nm) by •OH produced by QC, QCD or QCDA under the addition of H_2_O_2_ and acid with or without GSH. In brief, nine groups of experiments were prepared as follows and incubated at 37 °C for 3 min in the dark to decompose H_2_O_2_: (A) 2 mL PBS containing 0.8 mg mL^−1^ TMB, 30 mM H_2_O_2_ and 1 mg mL^−1^ QC, pH = 7.4, (B) 2 mL PBS containing 0.8 mg mL^−1^ TMB, 30 mM H_2_O_2_ and 1 mg mL^−1^ QCD, pH = 7.4, (C) 2 mL PBS containing 0.8 mg mL^−1^ TMB, 30 mM H_2_O_2_ and 1 mg mL^−1^ QCDA, pH = 7.4, (D) 2 mL PBS containing 0.8 mg mL^−1^ TMB, 30 mM H_2_O_2_ and 1 mg mL^−1^ QC, pH = 5.0, (E) 2 mL PBS containing 0.8 mg mL^−1^ TMB, 30 mM H_2_O_2_ and 1 mg mL^−1^ QCD, pH = 5.0, (F) 2 mL PBS containing 0.8 mg mL^−1^ TMB, 30 mM H_2_O_2_ and 1 mg mL^−1^ QCDA, pH = 5.0, (G) 2 mL PBS containing 0.8 mg mL^−1^ TMB, 30 mM H_2_O_2_, 10 mM GSH and 1 mg mL^−1^ QC, pH = 5.0, (H) 2 mL PBS containing 0.8 mg mL^−1^ TMB, 30 mM H_2_O_2_, 10 mM GSH and 1 mg mL^−1^ QCD, pH = 5.0, (I) 2 mL PBS containing 0.8 mg mL^−1^ TMB, 30 mM H_2_O_2_, 10 mM GSH and 1 mg mL^−1^ QCDA, pH = 5.0. Then, the absorbance of the supernatant obtained by centrifugation (13,000 rpm, 3 min) was tested by flow cytometry.

### 2.11. Photothermal Performance of QCDA

Totals of 1 mL of QC solution (0.5 mg/mL), 1 mL of QCD solution (0.5 mg/mL) and 1 mL of QCDA solution (0.5 mg/mL) were put into 1.5 mL centrifuge tubes, then exposed to laser irradiation (808 nm, 1.5 Wcm^−2^, 7.5 min) by power-tunable infrared laser (LR-MFJ-808/5000 mW, Changchun Lairui Photoelectric Technology Co., Ltd., Changchun, China). The real-time thermal images and the corresponding temperature quantitative value were determined by an infrared thermal camera (A35, FLIR Systems, Inc., Täby, Sweden).

### 2.12. Cytotoxicity Assays

The B16F10 cells and HepG2 cells were purchased from the National Collection of Authenticated Cell Cultures and cultured in Dulbecco’s modified eagle medium (DMEM; Gibco, Beijing, China), which was supplemented with 10% newborn bovine serum (NBS; Every Green, Huzhou, China) and 1% penicillin–streptomycin (Sangong Biotech, Shanghai, China) and incubated at 37 °C, 5% CO_2_. 

The B16F10 cells and HepG2 cells were planted separately in 96-well plates with a density of 7000 cells per well and incubated for 12 h. Then, different groups (Quercetin, QC and QCD) were added to the fresh, complete medium according to the gradient concentrations of 4, 8, 16, 32 and 50 μg/mL. The sample-free cell culture plate was used as a control. After 24 h of incubation, the medium was removed and 100 μL of MTT solution (0.5 mg/mL) was added to each well for another 4 h incubation. Then, the MTT solution was replaced with 100 μL of DMSO. After 15 min in a shaker at 37 °C, the absorbance at 490 nm was read by a multifunctional enzyme labeling instrument (Synergy H4, Bio-Tek, Winooski, VT, USA). For the QCDA group, all procedures were the same as above except for an additional 5 min of laser irradiation exposure (808 nm, 1.5 Wcm^−2^).

### 2.13. Cell Death Level Assay

A total of 4 × 10^5^ HepG2 cells were seeded into 6-well plates and incubated at 37 °C, 5% CO_2_ for 24 h. Then, the media were replaced with 2 mL of DMEM containing Quercetin, QC, QCD and QCDA at a concentration of 32 μg/mL. For the group QCDA + Light, the HepG2 cells were treated with QCDA for 4 h and then exposed to light for 10 min. After a total of 24 h, both adherent cells and cells suspended in the medium were collected and mixed before being washed three times with PBS. At last, the cells were incubated with Calcein-AM and PI staining reagents (Yeasen Biotechnology (Shanghai) Co., Ltd., Shanghai, China) for 15 min for the observation on a CLSM. The live cells were stained with green fluorescence, and dead cells as red fluorescence.

### 2.14. Cellular Uptake of QCDA In Vitro

The HepG2 cells were seeded at a density of 1 × 10^5^ cells into CLSM dishes and 6-well plates for 24 h. Then, the media were removed and the fresh DMEM containing QCDA (16 μg/mL) was added to the dishes for 2, 4 and 8 h. For the dishes, the media were sucked out, and the dishes were rinsed gently with phosphate-buffered saline (PBS) three times following fixation in 4% paraformaldehyde (PFA; Shanghai Macklin Biochemical Technology Co., Ltd., Shanghai, China) for 15 min and staining with 100 μL of 2-(4-Amidinophenyl)-6-indolecarbamidine dihydrochloride (DAPI; Beyotime, Shanghai, China) for 25 min at 37 °C. After being washed three times with PBS, the cellular uptake of QCDA was observed by a confocal laser scanning fluorescence microscope (CLSM; NIKON A1, Nikon Corporation, Tokyo, Japan). The 6-well plates were washed with PBS three times after removing the media. Then, 300 μL of trypsin (Gibco, Beijing, China) was added into the well for the collection of cells. After being washed three times with PBS, the cellular uptake of QCDA was observed via flow cytometry (Cytoflex A00-1-1102, Beckman Coulter, Suzhou, China).

### 2.15. The Mechanism of Cellular Uptake

The mechanism of cellular uptake was further investigated. The HepG2 cells were cultured on a six-well plate for 24 h at a density of 3 × 10^5^ cells, and then were pretreated for 1 h with different uptake inhibitors, including either 10 mg/L chlorpromazine, 1 mg/L indomethacin, 40 mg/L colchicine or 200 µM genistein. After that, the medium containing QCDA displaced the pretreated medium and continued to culture for 4 h. The fluorescence intensity was measured by flow cytometry.

### 2.16. ROS Generation Test In Vitro

The HepG2 cells were planted at a density of 1 × 10^5^ cells into CLSM dishes and cultured for 24 h. Then, the media in the dishes were removed before 1 mL of DMEM containing Quercetin, QC, QCD and QCDA at concentrations of 32 μg/mL was added. After 4 h of incubation, the HepG2 cells were lightly washed with PBS three times and incubated with DCFH-DA solution for 30 min. After fixation with 4% paraformaldehyde and staining of the nuclei with DAPI, the production of ROS was observed on a CLSM. For group QCDA + Light, the HepG2 cells were treated with QCDA for 4 h and then exposed to light for 20 min.

## 3. Results

### 3.1. Characterization of Quercetin, QC, QCD and QCDA

**Figure 1:**
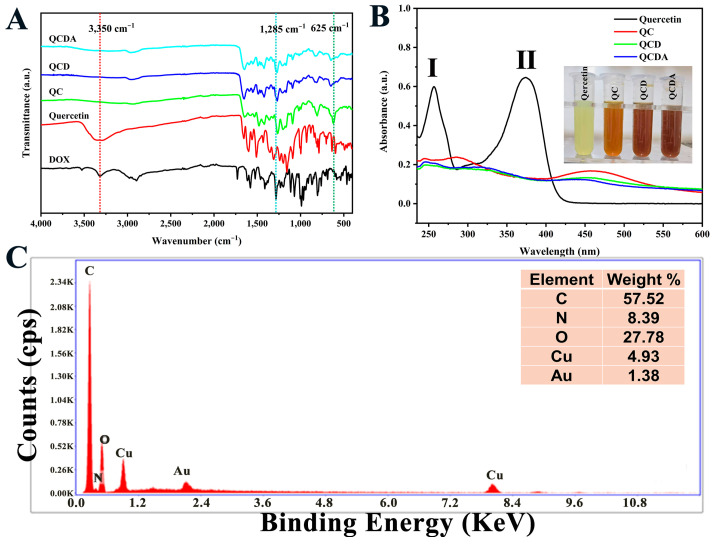
(**A**) The FT-IR spectrum of DOX, Quercetin, QC, QCD and QCDA. (**B**) The UV Spectrum of Quercetin, QC, QCD and QCDA, bands I and II corresponding to the two major absorption bands of Querrcetin. (**C**) The EDS smart quant results of QCDA.

In this study, Quercetin and Cu^2+^ were chosen to construct a functional carrier for the delivery of DOX and the photothermal agent (Au NPs) through a simple “all in one” strategy. Compared with Quercetin, the obvious rising FT-IR absorption peak of QC at 625 cm^−1^ (Figure 1A) could be attributed to the stretching vibration of Cu=O, which confirmed the formation of the QC coordination compound [34]. The FT-IR shoulder peak at 1285 cm^−1^, which belonged to C-H bending on the mono-oxygen six-membered ring of DOX [42], implied that DOX could be successfully loaded onto QC. As shown in Figure 1B, Quercetin exhibited two major absorption bands (bands I and II) in the UV/visible region. Compared with Quercetin, QC produced bathochromic shifts in the absorbances of bands I and II, which indicated the coordination between Quercetin and Cu ions [43]. The FT-IR and UV spectrum of QCDA had almost no change compared with QCD, which indicated that the structure of QCD had not been damaged in the process of loading Au NPs.

### 3.2. EDS Analysis of QCDA

In order to determine the surface chemistry of QCDA, EDS analysis was performed (Figure 1C). Combined with the FT-IR and UV spectrum, the presence of C, O and Cu further proved that Quercetin was coordinated with Cu. The EDS result also confirmed that Au NPs were loaded onto QCD successfully and the loading capacity was about 1.38%.

### 3.3. The Size, Zeta-Potential and Morphology of QC, QCD and QCDA

**Figure 2:**
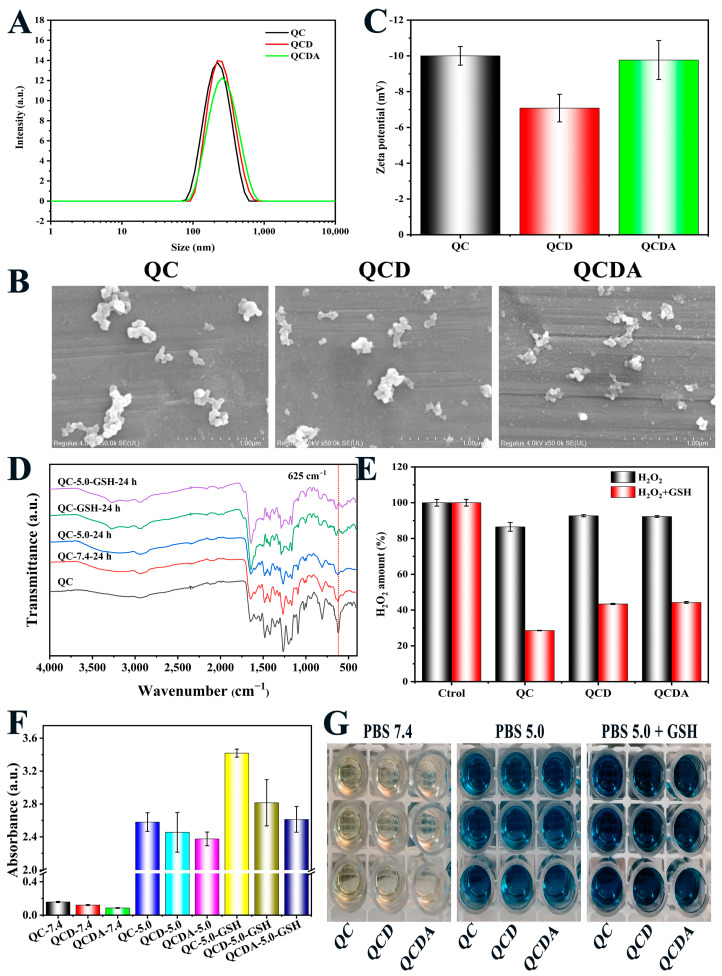
The particle size distribution (**A**), SEM images (**B**) and zeta potentials (**C**) of QC, QCD and QCDA. (**D**) The FT-IR spectrum of QC treated with GSH. (**E**) H_2_O_2_ consumption by QC, QCD and QCDA in the presence of GSH. The UV absorbance of TMB solutions with the selected conditions in the presence of H_2_O_2_ (**F**) and the corresponding photos (**G**).

The particle size distribution of QC, QCD and QCDA were analyzed by DLS at first. As shown in Figure 2A, the hydrodynamic diameter of QC and QCD were 233 ± 5.2 nm and 231 ± 3.5 nm, which were suitable for the biological applications [44,45,46]. The hydrodynamic diameter of QCDA (256 ± 6.8 nm) was slightly larger than QCD, which was mainly due to the loading of Au NPs. The morphology of QC, QCD and QCDA were all spheroidicity (Figure 2B), and the sizes were about 95 ± 7.3, 55 ± 5.9 and 70 ± 4.2 nm, respectively. The SEM images also showed a nanoparticle aggregation that may be due to the binding properties of PVP and the size of the nanoparticle aggregation matched well with the hydrodynamic diameter, which indicated that QC, QCD and QCDA were uniformly dispersed in the water in a state of nanoparticle aggregation. Compared with QC (−10 ± 0.5 mV), the increased zeta potential of QCD (−7.1 ± 0.8 mV) was mainly caused by the loading of DOX (Figure 2C). The reduced zeta potential of QCDA (−9.8 ± 1.1 mV) indicated that the Au NPs were being loaded, which was consistent with DLS and SEM results.

### 3.4. pH/GSH Response and H_2_O_2_ Decomposition Ability of QC, QCD and QCDA

The stability of QC under different pH values was studied and the results are shown in Figure 2D. Compared with the untreated QC, the FT-IR absorption peaks of QC treated with 7.4 PBS and 5.0 PBS solution at 625 cm^−1^ (attributed to Cu=O) were gradually decreasing, indicating that the acidic microenvironment could have a slight effect on the stability of QC. After being treated with GSH, the FTIR absorption peak at 625 cm^−1^ obviously disappeared, which clarified that QC had a more sensitive GSH-responsive decomposition ability, possibly as a result of the reduction in Cu^2+^ by GSH. Therefore, the H_2_O_2_ decomposition ability of QC, QCD and QCDA were tested both with and without GSH. As shown in Figure 2E, no more than 10% of H_2_O_2_ can be consumed in 30 min by QC, QCD and QCDA without GSH. Encouragingly, more than 60% of H_2_O_2_ could be decomposed within 30 min at 10 mM GSH thanks to the Fenton catalyst Cu^+^ reduced by GSH. 

However, Quercetin is a typical multi-phenol compound with antioxidant effects [47,48,49], which may deplete ROS and thus negate the effectiveness of CDT. Therefore, the amount of ROS produced by QC, QCD and QCDA under different conditions was studied using 3,3′,5,5′-tetramethyl-benzidine (TMB) for colorimetric detection of •OH species, during which the colorless TMB can be oxidized to chromogenic TMB cation-free radicals with the characteristic absorbance at 652 nm. As shown in Figure 2F,G, all three groups produced almost no ROS under neutral conditions. However, large amounts of ROS could be detected for the three groups under acidic conditions, suggesting that the antioxidant effect of Quercetin was insufficient to inhibit the effect of CDT. In the presence of GSH, the production of ROS in the three groups increased further, which may be due to the increase in the Fenton catalytic center caused by GSH reduction in Cu^2+^ to Cu^+^. The ROS produced by QCD and QCDA gradually decreased compared with QC, which may be caused by the reduction in copper ion content due to the loading of DOX and Au NPs. The production of ROS and the depletion of GSH could amplify oxidative stress, resulting in improved ROS-mediated chemodynamic therapy.

### 3.5. The Drug Loading Efficiency and In Vitro Drug Release Study

**Figure 3:**
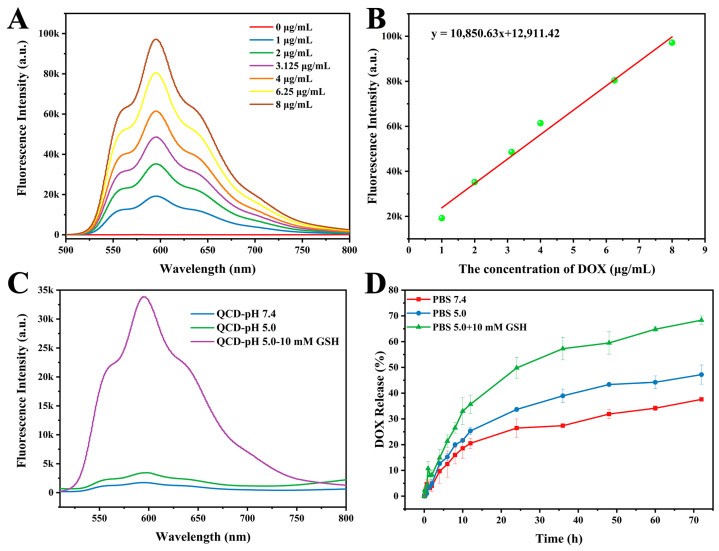
The fluorescence spectra (**A**) excited by 480 nm and the corresponding standard curve (**B**) of DOX. (**C**) The fluorescence spectra of QCD treated with 7.4, 5.0 and 5.0 PBS with 10 mM GSH. (**D**) The drug release curves of QCD treated with 7.4 and 5.0 PBS with or without 10 mM GSH.

In order to calculate the drug loading efficiency (DL), the fluorescence intensity of solutions with different DOX concentrations was detected by a spectrofluorophotometer, and the excitation wavelength was 480 nm, as shown in Figure 3A. Then, the corresponding standard curve was fitted based on the fluorescence intensity at 595.5 nm and the drug concentration, as shown in Figure 3B. The DL was about 3.86%, calculated based on the standard curve. Subsequently, the responsive release of QCD was first detected by a spectrofluorophotometer, as shown in Figure 3C.

Compared with the supernatant of QCD treated with 7.4 PBS, the fluorescence intensity of the supernatant, which QCD treated with 5.0 PBS, was only slightly enhanced. However, the fluorescence intensity of the supernatant of QCD treated with 5.0 PBS containing 10 mM GSH increased significantly, which indicated that DOX was released in response to GSH. Above all, the stimulus-response of QCD was mainly derived from the reducing stimulus of GSH, which matched well with the result of Figure 2D. Meanwhile, the release behaviors of QCD were evaluated in 7.4 PBS solution and 5.0 PBS with or without 10 mM GSH (Figure 3D). The DOX release of QCD treated with PBS 5.0 was slightly higher than that with PBS 7.4, which matched well with the FTIR results. However, the cumulative release of DOX in QCD treated with 5.0 PBS containing 10 mM GSH reached about 68% at 72 h, which was much higher than QCD treated with 7.4 PBS (about 37%) and 5.0 PBS (about 47%). It was shown that QCD had an obvious GSH responsive release behavior for anticancer and can remain relatively stable in normal tissues. 

### 3.6. The Photothermal Ability of QCDA

**Figure 4:**
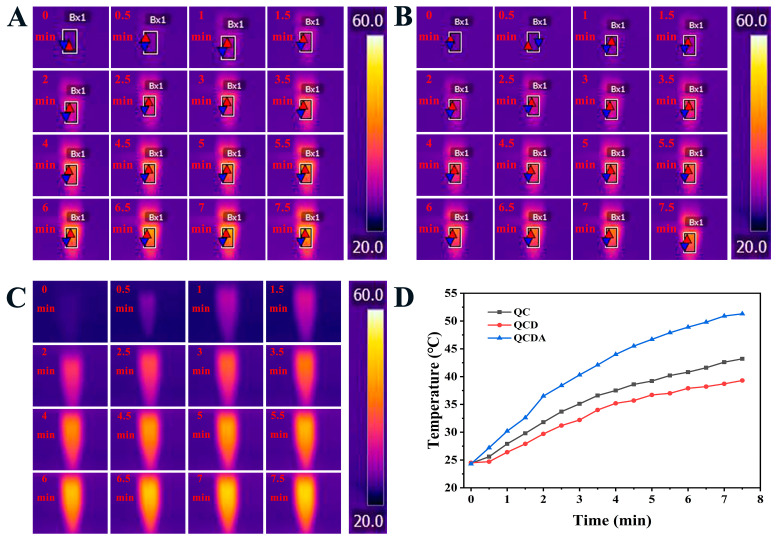
Thermographic images of different time points of QC (**A**), QCD (**B**) and QCDA (**C**) over a period of 7.5 min of laser irradiation (808 nm, 1.5 W cm^−2^). (**D**) The photothermal curve of QC, QCD and QCDA.

Au NPs, as a kind of excellent photothermal conversion agent, have attracted widespread attention in PTT. Therefore, the photothermal performance of QCDA was explored under laser irradiation (808 nm, 1.5 Wcm^−2^, 7.5 min) by a power-tunable infrared laser. In addition, the photothermal property of QC and QCD were explored for comparison. As shown in Figure 4A,D, QC exhibited certain photothermal properties mainly due to the broad absorptions in the NIR window of QC [34,50]. Then, the photothermal performance of QCD was reduced compared with QC (Figure 4B,D), which may be caused by the loading of DOX. With the loading of Au NPs, QCDA exhibited enhanced photothermal performance due to the superior photothermal conversion ability of Au NPs (Figure 4C,D), which indicated that QCDA was an ideal platform for PTT.

### 3.7. In Vitro Cytotoxicity Assay

**Figure 5:**
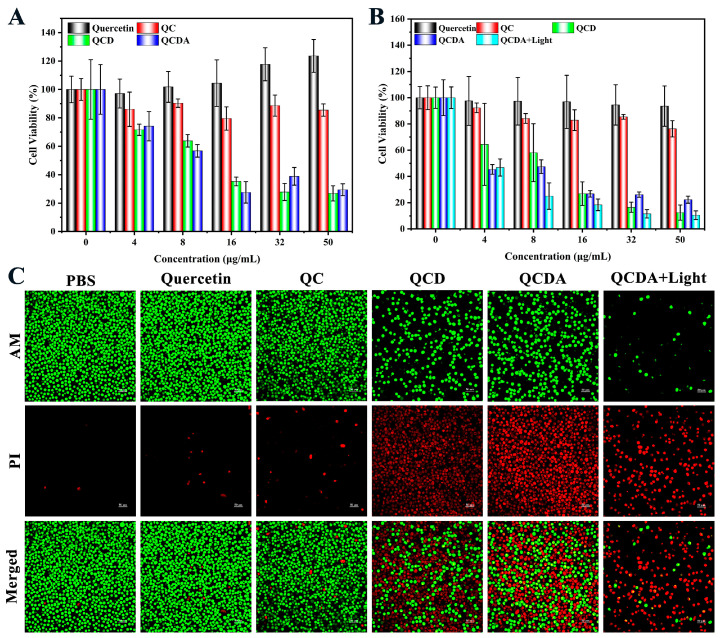
(**A**) Cell viability assay of B16F10 cells after different treatments. (**B**) Cell viability assay of HepG2 cells after different treatments. (**C**) Live and dead staining image of HepG2 cells incubated with PBS, Quercetin, QC, QCD, QCDA and QCDA + Light, (The live cells were stained with green fluorescence, and dead cells as red fluorescence.).

Based on the excellent physical and chemical properties of QCDA, the cytotoxicity assay toward B16F10 cells and HepG2 cells in vitro was performed by MTT. As shown in Figure 5A,B, all QC, QCD and QCDA exhibited dose-dependent cytotoxicity to both B16F10 cells and HepG2 cells. QC showed more cytotoxicity than Quercetin, possibly due to CDT being enhanced by the decomposition of excess H_2_O_2_ in cancer cells by Cu^+^ and the depletion of GSH by Cu^2+^. The cell’s viability was further reduced after the treatment of QCD, demonstrating that the loaded DOX maintained its significant cytotoxicity. The half-maximal inhibitory concentration (IC50) of QCDA was 3.46 ± 0.35 μg/mL to HepG2 cells, which was much lower than 11.466 ± 0.42 μg/mL to B16F10 cells. That meant under the same treatment conditions, QCD showed a stronger killing effect on HepG2 cells than B16F10 cells. Therefore, HepG2 cells were selected to verify the photothermal therapy of QCDA. Encouragingly, QCDA exhibited a stronger anticancer ability with laser irradiation than without irradiation, and the IC50 dropped to 2.838 ± 0.09 μg/mL, which may be due to the photothermal effect of Au NPs enhanced by PTT and CDT.

Live and dead staining images of HepG2 cells were captured by CLSM. The green fluorescent dots represent living cells, and the red fluorescent dots mean dead cells. However, all cells in the QCD, QCDA and QCDA + Light groups showed red fluorescence due to DOX loaded in QCD and QCDA. Therefore, the non-green dots in the merged images represent dead cells for the three groups. As shown in Figure 5C, HepG2 cells treated with QCDA under laser irradiation showed the best death outcomes, which matched well with the MTT results. Above all, we can infer that QCDA was a synergistic therapeutic platform integrating chemotherapy, chemodynamic therapy and photothermal therapy.

### 3.8. Cellular Uptake

**Figure 6:**
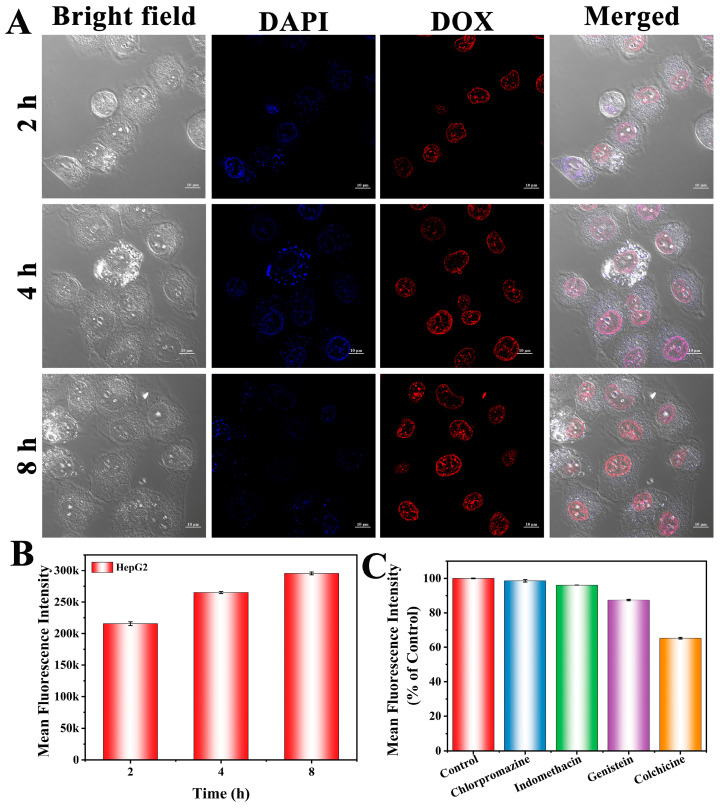
(**A**) CLSM images of HepG2 cells incubated with QCDA at different incubation times. (**B**) The fluorescence intensities of QCDA in HepG2 (*n* = 3). (**C**) Relative fluorescence intensities of HepG2 cells co-treated with QCDA and various uptake inhibitors (*n* = 3).

The intracellular uptake of QCDA after 2, 4 and 8 h incubation times were investigated by CLSM and flow cytometry. As shown in Figure 6A, it was observed that red fluorescence appeared in the cell nucleus at 2 h, which indicated that QCDA can be effectively uptaken by HepG2 cells in a short time. The red fluorescence intensity in the cell nucleus increased with the extension of uptake time, which proved that the QCDA could be gradually decomposed in HepG2 cells. Meanwhile, the intensity of red fluorescence (DOX) at 8 h was significantly stronger than that at 2 and 4 h, which indicated that the cellular uptake of QCDA was time-dependent (Figure 6B). The mechanism of cell uptake was revealed by the fluorescence ratio percentage of each inhibitor group to the control group. Chlorpromazine and genstein can separate clathrin and adaptin from cytomembrane to break the clathrin-mediated endocytosis. Colchicine interferes the macropinocytosis by blocking microtubule transport. Indomethacin inhibits caveolinmediated endocytosis. As illustrated in Figure 6C, QCDA was mainly internalized by HepG2 cells through macropinocytosis-mediated endocytosis.

### 3.9. Intracellular ROS Generation

**Figure 7:**
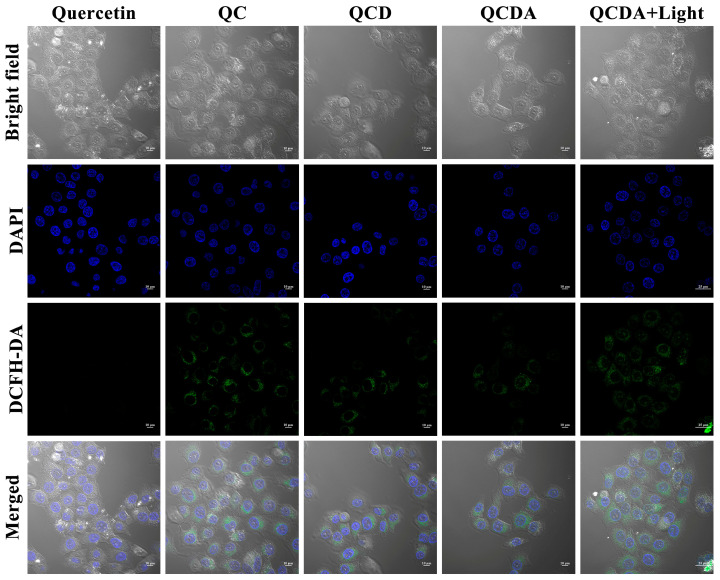
CLSM images of ROS in HepG2 cells incubated with Quercetin, QC, QCD and QCDA for 4 h.

The production of excess ROS in cancer cells was a necessary condition for chemodynamic therapy. To investigate the intracellular ROS generation of Quercetin, QC, QCD, QCDA and QCDA + Light, DCFH-DA was adopted as the ROS-specific probe. As shown in Figure 7, HepG2 cells incubated with QC showed obvious green fluorescence compared to Quercetin, which corresponded to an amount of ROS production. That might be attributed to the Fenton catalyst Cu^+^ reduced by GSH. At the same concentration of QC, QCD and QCDA, the green fluorescence of HepG2 cells treated with QCD and QCDA was slightly reduced than QC, possibly due to the reduction in Cu^2+/+^ content caused by the loading of DOX and Au NPs. However, under laser irradiation, the green fluorescence of HepG2 cells treated with QCDA was significantly stronger than that without light, which indicated that PTT enhanced the activity of the Fenton catalyst to strengthen CDT.

## 4. Conclusions

In summary, we constructed a GSH-responsive QCDA nanotheranostic platform, which was formed by the coordination of Quercetin and Cu^2+^, followed by the loading of DOX and Au NPs for synergistic anticancer therapy. Under the high GSH condition of TME, QCDA was decomposed to release Quercetin, DOX, Cu^+^ and Au NPs. Cu^+^ reduced by GSH catalyzed H_2_O_2_ to produce ROS, which caused severe oxidative damage to biomolecules and organelles for CDT. Meanwhile, the high concentration of GSH was rapidly oxidized to GSSG by Cu^2+^, which further disrupted the redox homeostasis of cancer cells. The released Au NPs could increase the temperature of cancer cells under near-infrared light irradiation, which not only caused cancer cell death of PTT but also accelerated Fenton catalytic activity for enhanced CDT. Quercetin and DOX can inhibit cancer cell growth and kill cancer cells through chemotherapy assisted by CDT and PTT. Taken together, this study indicated that the QCDA nanotheranostic platform holds great potential for synergistic photothermal-enhanced chemotherapy and chemodynamic therapy for anticancer.

## Data Availability

Data are available on request from the corresponding author.
